# Characteristics of viral ovarian tumor domain protease from two emerging orthonairoviruses and identification of Yezo virus human infections in northeastern China as early as 2012

**DOI:** 10.1128/jvi.01727-24

**Published:** 2024-12-31

**Authors:** Zi-Yun Chen, Jie Zhang, Pei-Jun He, Tao Xiong, Dai-Yun Zhu, Wen-Jie Zhu, Xue-Bing Ni, Li-Feng Du, Qian Wang, Ya-Wei Zhang, Luo-Yuan Xia, Dong-Sheng Chen, Liang-Jing Li, Ming-Zhu Zhang, Xiao Ming Cui, Tian-Hong Wang, Juan Wang, Zhenfei Wang, Tian-Feng An, Wu-Chun Cao, Xiao-Hua Liu, En-Jiong Huang, Na Jia

**Affiliations:** 1 School of Public Health of Fujian Medical University 74551 , Fuzhou, Fujian, China; 2 State Key Laboratory of Pathogen and Biosecurity, Academy of Military Medical Sciences 602528 , Beijing, China; 3 School of Public Health and Health Management, Gannan Medical University 74554 , Ganzhou, Jiangxi, China; 4 State Key Laboratory of Emerging Infectious Diseases and Centre of Influenza Research, School of Public Health, University of Hong Kong 90397 , Hong Kong, China; 5 State Key Laboratory of Genetic Engineering, Ministry of Education Key Laboratory of Contemporary Anthropology, School of Life Sciences, Fudan University 517826 , Shanghai, Shanghai, China; 6 Department of Biochemistry and Molecular Biology, College of Basic Medicine, Hebei University of Chinese Medicine 441322 , Shijiazhuang, Hebei, China; 7 Department of Toxicology and Health Inspection and Quarantine, School of Public Health, Tianjin Medical University 659295 , Tianjin, Tianjin, China; 8 Fuzhou International Travel Healthcare Center, Fuzhou, China; University of North Carolina at Chapel Hill, Chapel Hill, North Carolina, USA

**Keywords:** orthonairovirus, tick-bite patients, retrospective surveillance, ovarian tumor-like cysteine protease

## Abstract

Emerging tick-borne orthonairovirus infections pose a growing global concern, with limited understanding of the viral ovarian tumor-like cysteine proteases (vOTUs) encoded by novel orthonairoviruses. These vOTUs, a group of deubiquinylases (DUBs), disrupt the innate immune response. Yezo virus (YEZV), a recently discovered pathogenic orthonairovirus, was first reported in Japan in 2021. In this study, we successfully isolated and identified YEZV and a new orthonairovirus, Jiànchuān tick virus (JCTV), from *Ixodes persulcatus* and *Haemaphysalis montgomeryi* ticks, respectively, in China. We found that the vOTU domains encoded by YEZV and JCTV exhibited both DUB and deISGylase activities, though with potentially less broad deISGylation compared to that of Crimean-Congo hemorrhagic fever virus (CCHFV) during natural infection. Phylogenetic analysis of global vOTUs, including 83 new sequences, revealed a high diversity of this domain. Interestingly, retrospective screening of tick-bite patients from 2012 to 2016 in northeastern China traced YEZV infections as far back as 2012, identifying four cases. Additionally, YEZV primarily infected *I. persulcatus* (31.4%) and *Dermacentor nuttalli* (10.5%) in northern China, while JCTV exhibited high infection rates in *H. montgomeryi* (81.3%) in southern China. In summary, our work emphasizes the active surveillance of orthonairovirus infections and the imperative need for the development of vOTU domain-targeted anti-virals, offering potential therapeutic solutions for a broad spectrum of orthonairoviruses.

**IMPORTANCE:**

The vOTUs, a group of DUBs, mimic the functions of host DUBs to enhance viral infectivity and may serve as potential drug targets. vOTUs from different orthonairoviruses exhibit distinct preferences toward ubiquitin (Ub) and ubiquitin-like protein interferon stimulated gene 15 (ISG15). In this study, we investigated the deubiquitinase and deISGylase functions of various orthonairoviral vOTUs using both an overexpression system and natural viral infections *in vitro*. Our findings illustrate that the vOTUs from YEZV and JCTV can cleave both Ub and ISG15 in an overexpression system, but these viruses exhibit potentially narrower deISGylation capacity than CCHFV during natural infection. This suggests that the diversity of vOTUs may have a potential relationship with the pathogenesis.

## INTRODUCTION

*Nairoviridae*, within the order *Bunyavirales*, are negative-sense single-stranded RNA viruses that infect various vertebrate species, including humans, primarily through tick transmission (1). In addition to Crimean-Congo hemorrhagic fever (CCHF), which is listed in the World Health Organization’s List of Blueprint Priority Diseases (2), recent discoveries highlight the emergence of novel orthonairoviruses capable of infecting humans. Among these newly identified pathogens are Yezo virus (YEZV) (3), Běijí nairovirus (4), Sōnglǐng virus (5), Tǎchéng tick virus 1 (6), and Wetland virus (7), which often cause febrile illness and, in some cases, lead to fatal outcomes. YEZV was first reported in 2021 in seven Japanese cases of acute febrile illness (3), and its non-specific clinical manifestations were subsequently described (8–10). However, the viral characteristics and pathogenesis of YEZV remain largely unknown.

Ovarian tumor domain proteases (OTUs), a group of deubiquinylases (DUBs), have been uncovered as key regulators of ubiquitin (Ub)-modified proteins in essential cellular processes and pathways (11). It is not surprising that viruses, including orthonairoviruses, have evolutionarily developed effector proteins that mimic host OTUs to suppress the host cell’s anti-viral responses (12). Despite the absence of licensed vaccines or specific anti-virals for nairoviral diseases, including CCHF, the development of anti-virals targeting orthonairoviral viral ovarian tumor-like cysteine proteases (vOTUs) has made these proteins attractive pharmacological targets (13). However, vOTUs originating from different orthonairoviruses exhibit distinct preferences for Ub and the ubiquitin-like protein interferon stimulated gene 15 (ISG15) (14). For instance, Dugbe virus shows a strong preference for Ub (15), while Erve virus displays much higher activity toward ISG15 substrates (14). In contrast, Crimean-Congo hemorrhagic fever virus (CCHFV) vOTU can cleave both Ub and ISG15 efficiently, displaying both DUB and deISGylase activities (16). These differences may influence the specificity of orthonairoviral vOTUs in pathogenesis and host preference (14). Thus, it is crucial to understand the target preferences of viral OTUs from newly identified pathogenic orthonairoviruses, such as YEZV, particularly in the context of viral infections, to support the potential viability of viral vOTUs as therapeutic targets for a broad spectrum of orthonairoviruses.

In this work, based on the successful isolation of YEZV and another new species of orthonairovirus (Jiànchuān tick virus [JCTV]) from ticks, we explored the DUB and deISGylase functions of various orthonairoviral OTUs both under an overexpression system and in natural viral infections *in vitro*. We demonstrated that the OTU domains encoded by YEZV and JCTV exhibited both DUB and deISGylase activity. However, these viruses exhibited potentially narrower deISGylation than CCHFV during natural infection. Additionally, through a retrospective screening of 346 tick-bite patients in northeastern China, 4 cases of YEZV infection were identified as early as 2012.

## MATERIALS AND METHODS

### Cell lines

Mammalian Vero 81 (American Type Culture Collection [ATCC], Cat. No. CCL-81), BHK-21 (ATCC, Cat. No. CCL-10), HuH7, THP-1 (ATCC, Cat. No. TIB-202), L929 (ATCC, Cat. No. CCL-1), HepG2 (ATCC, Cat. No. HB-8065), and 293T (ATCC, Cat. No. CRL-11268) were maintained in Dulbecco’s Modified Eagle Medium (Gibco). HuH7 cells were purchased from the Cell Bank of the Chinese Academy of Sciences (Shanghai, China). HUVEC (ATCC, Cat. No. PCS-100–00) and C6/36 (ATCC, Cat. CRL-1660) cell lines were maintained in Roswell Park Memorial Institute 1640 medium (Gibco), respectively. All were all supplemented with 10% fetal bovine serum (FBS, Gibco) at 37°C in a humidified atmosphere with 5% CO_2_. *Rhipicephalus microplus* BME/CTVM23 tick cells were maintained in L-15 (Leibovitz) medium supplemented with 20% FBS and 10% tryptose phosphate broth at 32°C (17). *Ixodes scapularis* tick cell line IDE8 was maintained in L-15B medium supplemented with 10% tryptose phosphate broth, 10% FBS, and 0.1% bovine lipoprotein (18).

### Tick orthonairovirus isolation

The −80°C stored tick samples were collected during a previous large-scale tick virome research (19). Approximately 400 ticks, primarily from 2022, were used for orthonairovirus isolation, with 10–15 ticks pooled for each isolation. Briefly, the ticks were ground in phosphate buffered saline (PBS) using a pestle in a porcelain mortar. The resultant suspensions were filtered through a 0.22 µm syringe filter, and then aliquots were inoculated onto monolayers of around 60% confluent cells, including Vero 81, BHK-21, HUVEC, IDE8, and CTVM23. After incubation at 28°C for 1 h, the cells were topped up with the respective growth medium (containing antibiotics) and maintained at 32°C for 7 days. The supernatants from all cell types in which isolation was attempted were then examined using a set of orthonairovirus primers (Table S1) with One Step TB Green PrimeScript RT-PCR Kit (TaKaRa). Three successive cell passages were tested.

### *In vitro* culture of YEZV or JCTV

YEZV was initially successfully cultured in Vero 81 cells, and JCTV was cultured in HUVEC cells. To determine viral growth in other cell lines, three replicate cultures of Vero81, BHK21, Huh7, HUVEC, C6/36, IDE8, CTVM23, HepG2, L929, and Thp-1 were infected with YEZV or JCTV (supernatant from the original cell culture in which the virus was isolated). After inoculation and incubation at 32°C for 2 h, the cells were washed; fresh medium was added, and the plates were incubated at 37°C for up to 8 days. Then, 100 µL of supernatant was collected from each culture at 24 h post-infection over 8 days’ time period. Quantitative TaqMan One-Step Real-time RT-PCR (TaKaRa) assays targeting YEZV or JCTV were used to quantify viral concentration (Table S1) following the manufacturer’s instructions and were repeated three times from each flask of infected cells. The copy numbers were calculated using a standard curve method using a linearized plasmid containing the corresponding segment.

The live virus in different cell cultures was quantified using the 50% tissue culture infective dose (TCID_50_) method. Cells were seeded in a 96-well plate and inoculated with serial dilutions of the virus. The cell plates were then incubated and examined for the virus-mediated cytopathic effect (CPE), and TCID_50_ per milliliter was calculated using the Reed-Muench method (20).

### Viral genome sequencing

Total RNA was extracted from Vero 81 cell supernatants containing YEZV or from HUVEC cells containing JCTV using a High Pure Viral RNA Kit (Roche, Switzerland). A high-throughput sequencing library was constructed using an Ion Total RNA-Seq Kit v.2 (Thermo Fisher Scientific, Massachusetts, USA) and deep-sequenced using an Ion Torrent Personal Genome Machine (Thermo Fisher Scientific). The sequenced reads were filtered using the NGS QC Toolkit v.2.3.36 to remove low-quality and short reads. The clean reads were assembled using Newbler v.2.9 (Roche). Assembled contigs were linked and extended to create a full-length sequence using Cytoscape v.2.8.3.

### Fluorescence *in situ* hybridization

YEZV or JCTV-infected BHK-21 cells were fixed in 3.7% (vol/vol) formaldehyde in PBS in wells of a 24-well plate for 10 min at room temperature, then immersed in 70% (vol/vol) ethanol for at least 1 h at 4°C to permeabilize the cells. To ensure both high sensitivity and high specificity, probe sets (Stellaris RNA FISH probes), comprising up to 48 singly labeled oligonucleotides for each set, were synthesized (Table S2) (Biosearch Technologies). Probe sets were labeled with Quasar 570.

### Immunofluorescence

The polyclonal antibodies against the specific peptides of the M segments of YEZV and JCTV were produced in rabbits using standard protocols (Sino Biological, China). YEZV- or JCTV-infected BHK-21 cells were fixed in acetone. Primary antibodies to YEZV or JCTV generated in rabbits were incubated with the fixed samples at 4℃ overnight. Antibody binding was detected using fluorescein isothiocyanate-conjugated anti-rabbit antibody (Abcam) at a 1:1,000 dilution in PBS-0.5% Tween 20. The samples were counterstained with the nuclear stain DAPI and viewed with an Olympus DP74 microscope (Japan).

### Transmission electron microscopy

Resuspended cells from the YEZV or JCTV-infected BHK-21 cell cultures were centrifuged at 800 × *g* for 5 min; the supernatant was discarded, and the cell pellet was fixed in 2.5% glutaraldehyde (wt/vol) for 2 h. The cells were then dehydrated with a graded series of ethanol at 50%, 70%, 90%, and 100% before being embedded in resin.

The fresh resin was used to embed pellets in molds and cured for 48 h at 60°C. Ultrathin serial sections (50–100 nm) were cut and collected on Formvar-coated copper grids. Grids were post-stained with 2% uranyl acetate for 15 min and lead citrate for 10 min. After washing with double-distilled water and drying on copper grids, pellets were viewed at 80 kV using an H7650 transmission electron microscope (Hitachi, Japan).

### Transfection and Western blot analysis

Plasmids expressing the OTU domain of YEZV or JCTV were synthesized (Sangon, China) and cloned into pcDNA3.1 containing a C-terminal HA-tag. For DUB assay, pcDNA3.1-Flag-Ub was used; for deISGylase activity, pcDNA3.1-V5-hISG15, pcDNA3,1-Ube1L, pcDNA3.1-UbcH8, and pcDNA3.1-Herc5 were used. In brief, 293T cells were 70%–80% confluent at the time of transfection. For DUB transfection at six wells, 1 µg pcDNA3.1-HA-OTU and 1 µg pcDNA3.1-Flag-Ub were incubated with Lipofectamine 3000 transfection reagent (Invitrogen). For deISGylase activity at six wells, 0.5 µg pcDNA3.1-HA-OTU, 0.5 µg pcDNA3.1-V5-hISG15, 0.25 µg pcDNA3,1-Ube1L, 0.25 µg pcDNA3.1-UbcH8, and 0.5 µg pcDNA3.1-Herc5 were incubated with Lipofectamine 3000 transfection reagent. The empty plasmid of pcDNA3.1 was used as a negative control. Ub and ISG15 conjugation levels were assessed 48 h post-transfection using Western blot analysis. Briefly, total protein was quantified by the bicinchoninic acid assay method. Equal amounts of 293T cells from the test and control groups were electrophoresed on a 4%–15% gradient SDS polyacrylamide gel, transferred to polyvinylidene fluoride membranes, and processed for immunoblotting. HA-OTU was visualized with rabbit anti-HA (Cell Signaling Technology); V5-hISG15 was detected with anti-V5 (Abcam); Flag-Ub was used with anti-Flag (Abcam); and endogenous ISG15 was used with anti-ISG15 (Santa Cruz). Endogenous Ub was detected with anti-Ub or K48 and K63 poly-Ub (Cell Signaling Technology). Anti-Ube1L, anti-UbcH8, and anti-Herc5 were used to detect E1, E2, and E3 (Abcam). Bound antibodies were detected using horseradish peroxidase-conjugated goat anti-rabbit secondary antibodies (Abcam). The immunoblots were developed using a SuperSignal West Dura Extended Duration Substrate (Thermo Fisher Scientific). Immunoblot films were scanned into jpeg format using a scanner (Tanon-5200Multi, Tanon). Mouse tubulin and HRP-conjugated goat anti-mouse secondary antibody (Sigma–Aldrich) served as the positive control in the immunoblots.

### Phylogenetic tree

Amino acid sequences of L, M, and S segments of JCTV and YEZV in this study were aligned with those of the previously reported orthonairoviruses using the E-INS-I algorithm implemented in MAFFT v.7.3 (21). Predicted viral proteins of OTU domains were clustered into a set of “non-redundant” representative sequences with the threshold of 100% similarity using CD-HIT v.4.8.1 (22). The longest representative sequence for each cluster was aligned with downloaded references using the E-INS-I algorithm implemented in MAFFT v.7.3 (21). The IQ-Tree v.16.1 algorithm was used to determine the best-fit amino acid substitution model on the basis of each multiple sequence alignment, and the maximum-likelihood phylogenetic tree for L, M, and S segments and for OTU domains was assessed with bootstrap tests (1,000 replicates), respectively.

### Retrospective surveillance in a sentinel hospital

We conducted a retrospective study at Mudanjiang Forestry Central Hospital, Heilongjiang province, in northeastern China, where various emerging tick-borne diseases have been reported since 2012 (23–26). Paired serum samples (acute phase and convalescence phase) from tick-bite patients were collected and stored at −40°C from 2012 to 2016. Due to the hospital reconstruction and the coronavirus disease 2019 pandemic, we did not collect samples after 2016. Medical history and demographic information were recorded each year. Tick-borne pathogens, such as *Anaplasma phagocytophilum*, *Anaplasma capra*, *Candidatus Neoehrlichia mikurensis*, *Ehrlichia chaffeensis*, spotted fever group rickettsiae, *Borrelia burgorferi sensu lato*, *Borrelia miyamotoi*, *Babesia*, tick-borne encephalitis virus, Jingmen tick virus, and severe fever with thrombocytopenia syndrome virus (SFTSV), were recorded or identified by previous testing (26).

### Neutralization assay

HepG2 cells were seeded in 96-well plates and incubated overnight at 37°C under 5% CO_2_ to establish a monolayer. Serial twofold diluted serum, starting at 1:20, was incubated with 200 TCID_50_/100 µL of the virus for 5 days at 37°C. Fresh media were used as a negative control, and pure virus in culture media was used as a positive control. The cytopathic effects were observed, and the serum neutralizing antibody titers were calculated.

## RESULTS

### Isolation and characterization of a novel nairovirus (JCTV) and YEZV in China

In our prior investigation, we analyzed meta-transcriptomes derived from 31 diverse tick species collected from 148 sites across 30 provinces in mainland China. This comprehensive examination led to the identification of 724 RNA viruses belonging to 59 established RNA viral families and 7 putative “superclades” (19). In the present study, we focused on the family *Nairoviridae*, from which we retrieved 83 newly identified orthonairoviral sequences, encompassing complete or nearly complete L segments. We then designed primers targeting six known pathogenic orthonairoviruses and three newly discovered orthonairoviruses, selected based on their highest abundance in our tick transcriptomic sequences (see Table S1). Utilizing RNA-seq contigs assigned to *Nairoviridae*, we strategically chose around 400 archived tick samples for orthonairovirus isolation from 19 sites across 10 provinces (refer to Fig. S1). Ultimately, we successfully isolated two orthonairoviruses from two distinct collection sites.

**Fig 1:**
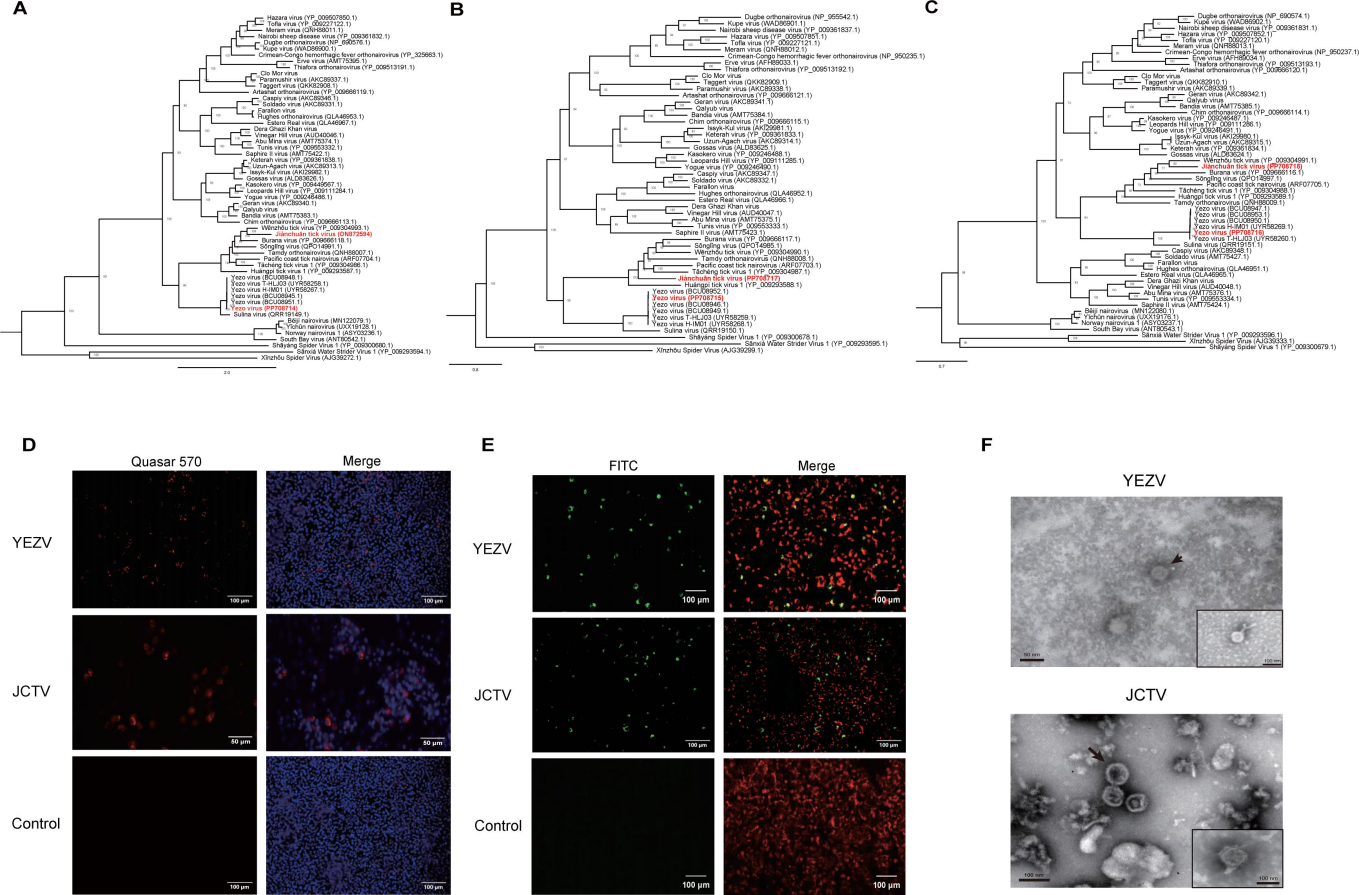
Characteristics of YEZV and JCTV detected in this study. (**A–C**). The phylogenetic trees of YEZV and JCTV based on L (**A**), M (**B**), and S (**C**) segments. The trees were constructed using the maximum-likelihood method with 1,000 bootstraps. The scale bar shows estimated evolutionary distance. (**D and E**) Fluorescence *in situ* hybridization (**D**) and indirect immunofluorescence assay (**E**) were used to detect YEZV and JCTV in BHK-21 cells, respectively. (**F**). Transmission electron microscopy showed YEZV and JCTV (arrows) in the cytoplasm of infected BHK-21 cells. Insets show negatively stained virions.

The complete genomes of the two orthonairoviruses were acquired through high-throughput sequencing of positive cell cultures. One full-genome sequence exhibited 97.8% identity with the Japanese YEZV, while the other displayed a 63.2% identity with the Wēnzhōu tick virus. The latter was designated as JCTV. YEZV was isolated from *Ixodes persulcatus* ticks in the Great Xing’an Mountain region of Heilongjiang province in northern China, while JCTV was isolated from *Haemaphysalis montgomeryi* ticks in Jiànchuān county, located in Yunnan province in southern China. Phylogenetic analysis of L and S segments indicated that YEZV grouped with Sulina virus, while JCTV was closely related to Wēnzhōu tick virus, as depicted in Fig. 1A through C. Fluorescence *in situ* hybridization (FISH) utilizing M segment-specific probe sets (see Table S2) revealed infection by JCTV and YEZV in the cells, as depicted in Fig. 1D. Subsequently, indirect immunofluorescence assay (IFA) further demonstrated their infections by employing M segment-specific antibodies against both viruses, as illustrated in Fig. 1E. Negatively stained electron microscopy demonstrated that both JCTV and YEZV presented as generally enveloped spheres. JCTV virions exhibited diameters ranging from 80 to 120 nm, while YEZV virions had diameters ranging from 60 to 180 nm. Transmission electron microscopy revealed the scattered presence of both YEZV and JCTV in the cytoplasm of the affected cells, as shown in Fig. 1F.

**Fig 2:**
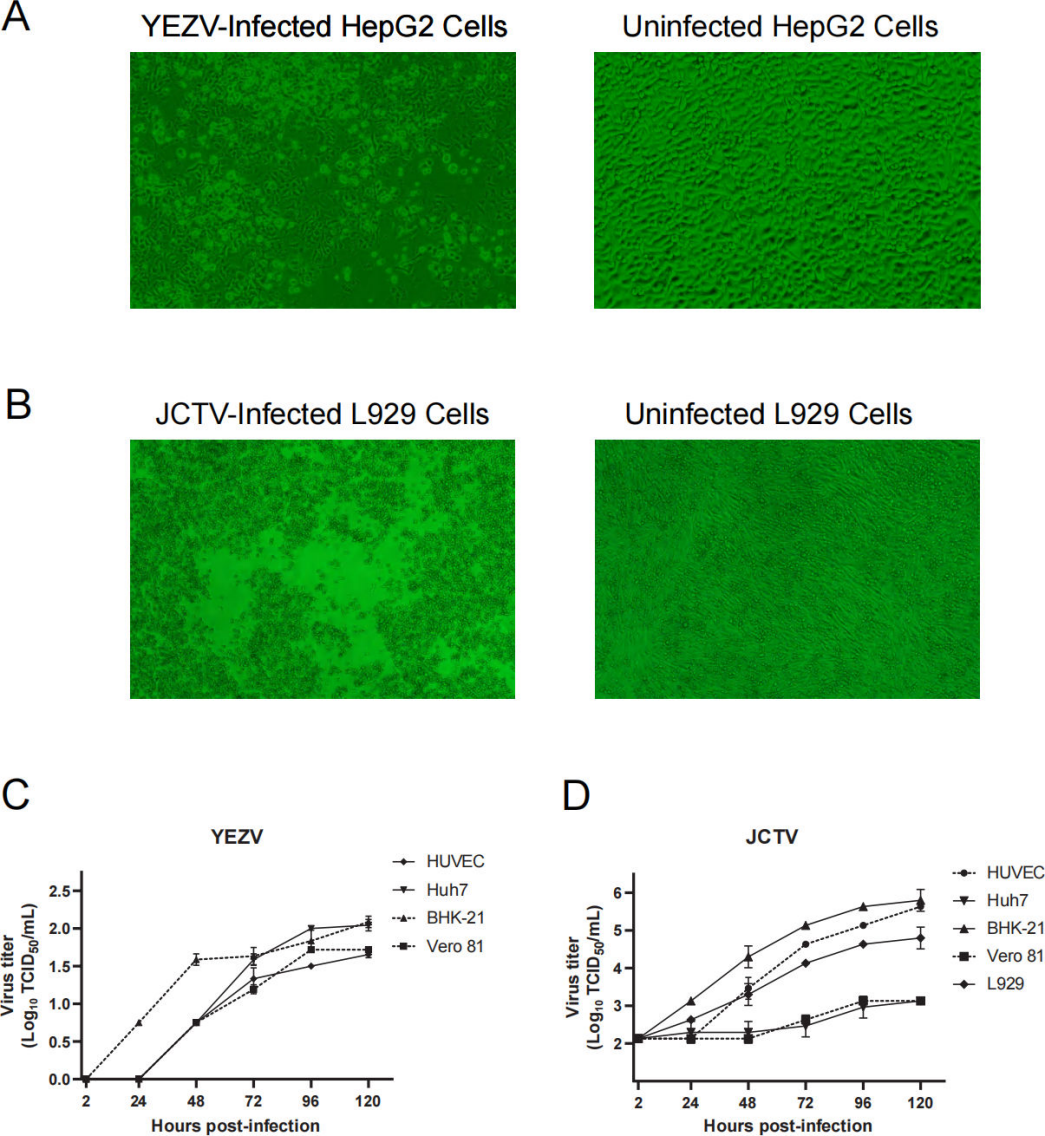
YEZV and JCTV infections in various cells. Light microscopy shows virus-induced cellular changes (cytopathic effect) in YEZV-infected HepG2 cells (**A**) and JCTV-infected L929 cells (**B**). Growth curves of YEZV (**C**) and JCTV (**D**) in HUVEC, BHK-21, Vero 81, Huh7, and L929 cells over 120 h, respectively. Titers of progeny viruses in the supernatants were measured by TCID_50_ assay. Error bars represent the standard deviation of the mean.

Virus growth was initially assessed by measuring viral genome copy concentrations in seven mammal cell lines (Vero 81, BHK-21, HuH7, L929, HepG2, Thp1, and HUVEC) and three arthropod cell lines (C6/36, IDE8, and CTVM23) (Fig. S2). YEZV could grow on BHK21, Huh7, Vero81, HUVEC, HepG2, and IDE8 cells, while JCTV growth was observed on BHK21, Huh7, L929, Vero81, and HUVEC cells, respectively, using reverse transcription quantitative PCR (RT-qPCR) (Fig. S2). Given that the infectious viral particles should be determined through live virus detection, we then quantified the viral growth and activity by the method of TCID_50_. For YEZV, obvious CPE was visualized only in HepG2 cells and only when a high concentration of virus (viral copy number higher than 10^7.3^ copies/μL) was inoculated, and for JCTV, obvious CPE was only observed in L929 cells (viral copy number higher than 10^2.8^ copies/μL) (Fig. 2A and B). No observable CPE was detected in the remaining eight cell lines. Therefore, we used HepG2 and L929 cells to evaluate the live viruses. YEZV demonstrated continuous viral replication on BHK21, Huh7, Vero81, and HUVEC cells, and JCTV exhibited live virus growth on BHK21, Huh7, L929, Vero81, and HUVEC cells (Fig. 2C and D).

### The effects of CCHFV, YEZV, and JCTV vOTU Overexpression on DUB and deISGylase Activity

**Fig 3:**
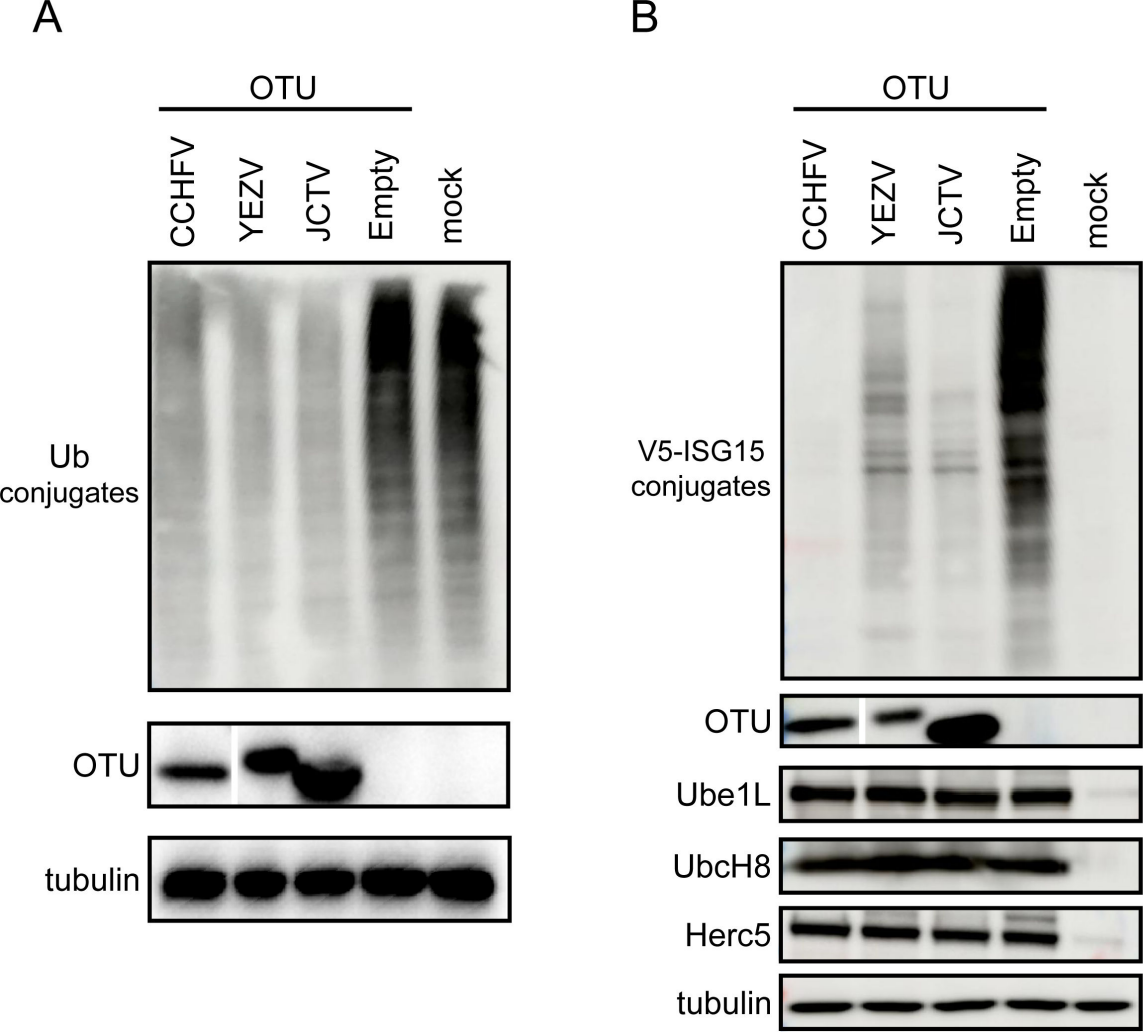
vOTU activity in transfected cells. (**A**) DUB activity of the vOTU of CCHFV, YEZV, and JCTV, respectively. 293T cells were co-transfected with plasmids expressing vOTUs and Ub. (**B**) DeISGylase activity of the vOTU of CCHFV, YEZV, and JCTV, respectively. 293T cells were co-transfected with plasmids expressing vOTUs, ISG15, Ube1L, UbcH8, and HERC5. Cell lysates were harvested 2 days post-transfection, and proteins were analyzed for Ub or ISG15-conjugates.

To assess whether the vOTU of YEZV and JCTV exhibit DUB and deISGylating activity, we transfected 293T cells with plasmids encoding the vOTU domains of CCHFV, YEZV, and JCTV. Remarkably, all three vOTUs demonstrated the ability to decrease the overall expression of Ub-conjugated proteins (Fig. 3A). To evaluate the impact of orthonairoviral vOTU expression on protein ISGylation, we generated ISG15 conjugates by co-transfecting plasmids expressing ISG15 and its specific enzyme (activating enzyme [E1, Ube1L] [27], conjugating enzyme [E2, UbcH8] [28], and ligase [E3, HERC5] [29]). Co-transfection of OTUs from CCHFV, YEZV, and JCTV resulted in a significant reduction in the levels of ISGylated proteins (Fig. 3B).

### General levels of ubiquitinated and ISGylated proteins in YEZV or JCTV naturally infected cells

**Fig 4:**
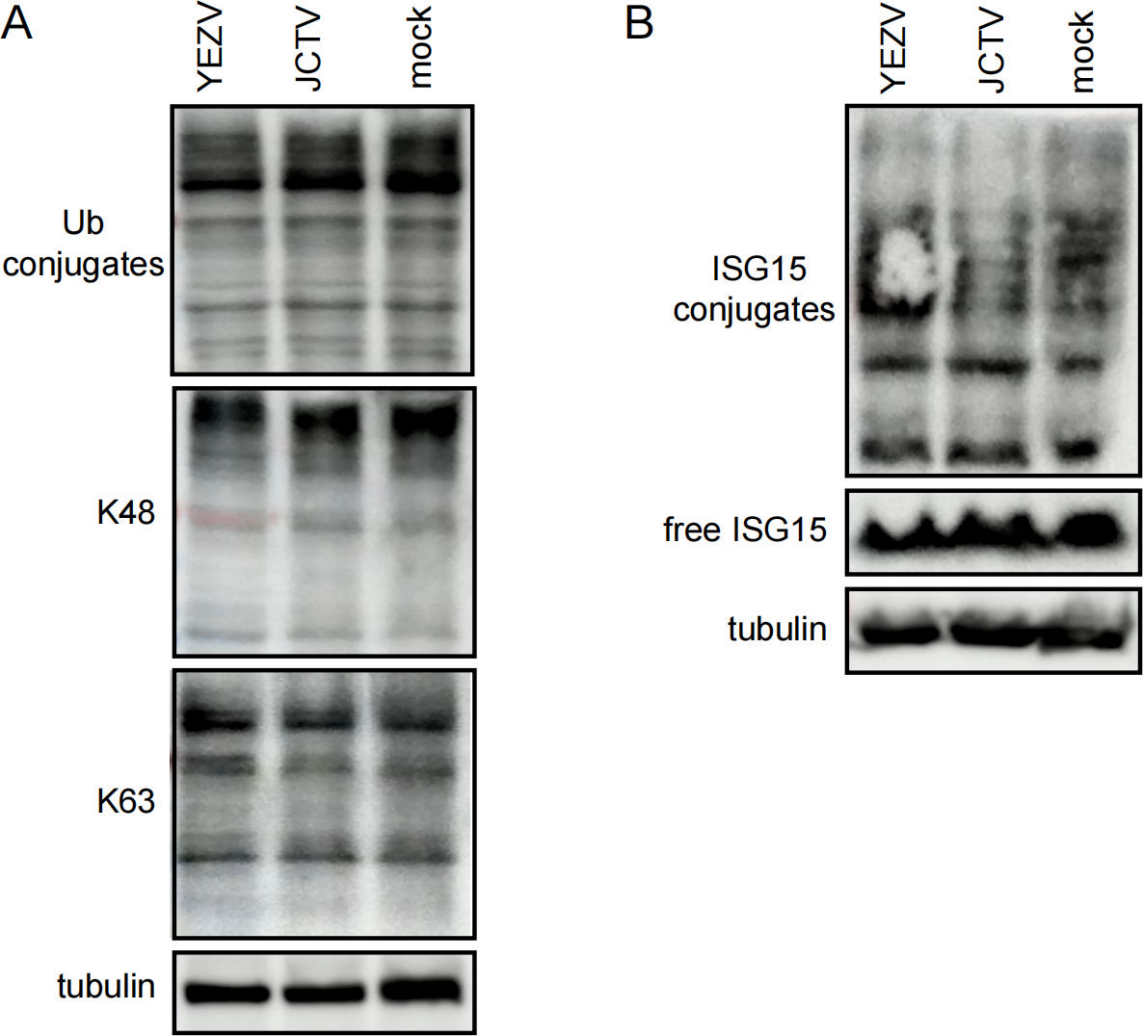
General levels of ubiquitinated/ISGylated proteins in Huh7 cells infected with YEZV and JCTV, respectively. Huh7 cells were infected with YEZV (1 × 10^8^ copies/μL) or JCTV (1 × 10^7^ copies/μL) viruses. (**A**) Western blot of ubiquitinated proteins in infected cells. Cell lysates were harvested 48 h post-infection, separated by SDS-PAGE, and probed for total Ub or K48- and K63-linked poly-Ub chains. (**B**) Western blot of ISGylated proteins in infected cells. Interferon-β (1.5 µg/mL) was added to the Huh7 cell culture media 4 h post-infection. Conjugation of ISG15 was visualized.

To further understand the function of vOTU on the levels of ubiquitinated and ISGylated proteins during natural viral infection with YEZV or JCTV, we infected Huh7 cells with YEZV (1 × 10^8^ copies/μL) or JCTV (1 × 10^7^ copies/μL). Visualization of total Ub-conjugated proteins by anti-Ub immunoblot showed no apparent differences between YEZV- or JCTV-infected and mock-infected cells. Similarly, the levels of K48- and K63-linked polyubiquitin chains, crucial in regulating the anti-viral innate immune response (30), exhibited no significant differences between YEZV- or JCTV-infected and mock-infected cells (Fig. 4A).

Subsequently, we investigated whether YEZV or JCTV viral infections possessed deISGylase activity. Interferon-treated Huh7 cells infected with YEZV or JCTV displayed robust protein ISGylation, suggesting restricted deISGylase activity by both viruses. Intriguingly, unlike CCHFV, which demonstrated broad deISGylase activity (16), YEZV and JCTV exhibited a more constrained pattern of deISGylation (Fig. 4B).

### Diversity of orthonairovirus vOTU

**Fig 5:**
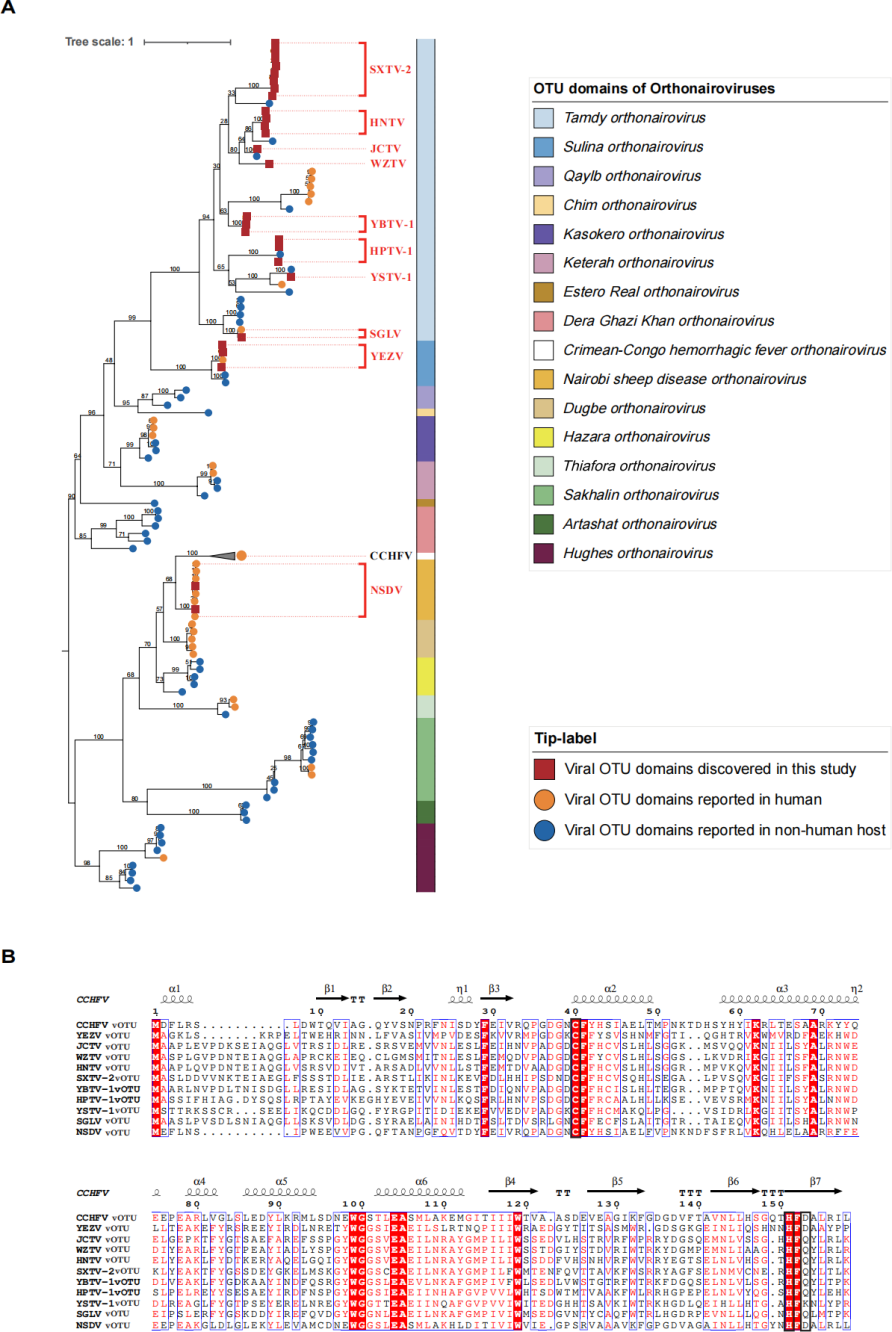
Phylogeographic analysis of diverse vOTU domains of orthonairoviruses. (**A**) The maximum-likelihood phylogenetic tree was built with vOTU domains from 93 global public sequences and 83 new ones from our work. vOTU domains were annotated in the Conserved Domains Database (https://www.ncbi.nlm.nih.gov/Structure/cdd/wrpsb.cgi). A total of 1,000 bootstraps were repeated to construct the tree. The scale bar shows estimated evolutionary distance. The orthonairoviruses of newfound sequences were indicated with red squares. The orthonairoviruses of public sequences that can infect humans are indicated with orange circles, and others are indicated with blue circles, respectively. Current species groupings are indicated by different colors, and the representative species are denoted. (**B**) The sequences of orthonairovirus vOTUs were visualized by ESPript 3.0. The catalytic triad is boxed in black. CCHFV, Crimean-Congo hemorrhagic fever virus; HNTV, Hénán tick virus; HPTV-1, Huángpí tick virus 1; JCTV, Jiànchuān tick virus; NSDV, Nairobi sheep disease virus; SGLV, Sōnglǐng virus; SXTV-2, Shǎnxī tick virus 2; WZTV, Wēnzhōu tick virus; YBTV-1, Yánbiān tick virus 1; YEZV, Yezo virus; YSTV-1, Yùshù nairo tick virus 1 .

Observing the significant role played by the vOTUs of YEZV and JCTV in viral DUB and deISGylase activity, we extended our analysis to understand the evolutionary patterns of the vOTU domain. Phylogenetic analysis, based on 93 public sequences and 83 new sequences from our study, revealed a Tamdy genogroup dominance in China (Fig. 5A). Additionally, potential new orthonairoviruses were identified, forming distant subclades related to known sequences, such as the new Shānxī tick virus 2 vOTU related to Burana virus, new Hénán tick virus vOTU related to Wēnzhōu tick virus, and new Yánbiān tick virus 1 vOTU related to Tamdy virus (Fig. 5A). Compared to the CCHFV, Nairobi sheep disease virus (NSDV), SGTV, and YEZV with known pathogenicity, the sequence diversity of these new orthonairoviral vOTU is shown in Fig. 5B. Each vOTU possesses a seven-stranded beta sheet as the core feature, with five major alpha helices framing the rest of the structure. The catalytic triads of CCHFV, YEZV, and NSDV are the conserved Cys-His-Asp motif, whereas Asp is replaced by Gln in most of new orthonairoviruses (Fig. 5B).

### Retrospective identification of tick-bite patients infected with YEZV but not JCTV as early as 2012

Within the scope of this study, a cohort of 346 tick-bite patients was enrolled. Serum samples from these individuals, featuring paired sera from both the acute and convalescent phases, were retrospectively examined using YEZV and JCTV antigen slides with the IFA method. Approximately 41.6% of the patients were admitted to the hospital in the year 2012. The median age of the 346 patients was 48 years, with an age range spanning from 4 to 81 years, and 50.1% were female. Tick bites were most frequently observed on the scalp, followed by the arms and legs. The time interval from the tick bite to the onset of illness ranged from 1 to 67 days, with a median of 4 days. A fever was reported in 29.9% of the patients.

In the final analysis, 4 of the 346 patients (1.2%) exhibited seroconversion of IgG antibodies against YEZV by IFA assays, as shown in Table 1 and depicted in Fig. S3. The neutralization assay was then performed on the four convalescent serum samples. The four serums all had a titer of 1:40 neutralization antibodies, indicating a low neutralizing protective activity. However, no positive reactions to JCTV were observed in any of the screened specimens. Notably, the positive serum samples were all collected in 2012, providing a temporal context to the emergence of YEZV. Unfortunately, detailed epidemiological and clinical information for these cases is lacking, representing a significant limitation in our understanding. The clinical symptoms of cases 1–3 were all mild (Table 1). Case 4, a previously healthy 49 year-old woman, experienced an 8 day tick bite on her left arm and exhibited a range of severe symptoms, including fever, dizziness, malaise, nausea, vomiting, and impaired right eye movement. Hematologic tests indicated leukocytosis, lymphocytopenia, thrombocytopenia, and elevated alanine aminotransferase. A severe optic nerve disorder was also observed, with abnormal cerebrospinal fluid (CSF) test results, revealing CSF lymphocytic pleocytosis. In addition, serum RNA from case 4 was positive to YEZV by quantitative RT-PCR with a copy number of 10^4.7^ copies/μL, while the other three cases were negative, possibly due to extremely low virus copy number after prolonged sample storage. Case 4 underwent treatment involving anti-viral medications and was discharged after a 14-day hospitalization period. Blood PCR for *Anaplasma*, *Ehrlichia*, *Borrelia*, *Rickettsia*, and *Babesia*, and RT-PCR for tick-borne encephalitis and Jingmen tick virus were all recorded as negative.

**TABLE 1:** Clinical characteristics and laboratory test results of the four patients, in 2012 from China^*a*^

Characteristics	Case 1	Case 2	Case 3	Case 4
Age (yr)	NA	50	59	49
Sex	Female	Female	Female	Female
Days between known tick bite and illness onset	NA	1	56	8
Elevated temperature (°C)	No	39	No	39
Dizziness	No	No	No	Yes
Malaise	No	No	No	Yes
Rash	No	Yes	Yes	No
Eschar	No	No	Yes	No
Lymphadenopathy	No	No	No	No
Nausea	No	No	No	Yes
Vomiting	No	No	No	Yes
Neck stiffness	No	No	No	No
Kernig’s sign	No	No	No	No
Optic nerve disorder	No	No	No	Severe
Hematologic test				
Leukocyte count (×10^−9^/L)	5.15	15.3	8.2	10.9
Lymphocyte (%)	53.21	9.2	29.2	19.4
Granulocyte (%)	35.72	90.5	66.7	77
Hemoglobin (g/L)	122	131	139	121
Platelet count (×10^−9^/L)	302	222	179	75
Biochemical tests				
AST (U/L)	NA	NA	18.5	23.6
ALT (U/L)	NA	NA	18.3	42.2
Cerebrospinal fluid measurements				
Leukocytes (×10^−9^/L)	NA	NA	NA	35

^*a*^ ALT, alanine aminotransferase; AST, aspartate aminotransferase; NA, not available (not performed or not reported).

### Tick surveillance for YEZV and JCTV distribution

Subsequently, a comprehensive surveillance was conducted to elucidate the epidemiological distribution of YEZV and JCTV in ticks. Additional 868 adult ticks sourced from nine sites across eight provinces were individually tested using qRT-PCR with specific primers for YEZV and JCTV. Seven dominant tick species, namely, *Ixodes ovatus*, *I. persulcatus*, *Amblyomma javanense*, *Haemaphysalis longicornis*, *H. montgomeryi*, *Dermacentor nuttalli*, and *Rhipicephalus microplus*, representing Northeast China, Central China, and Southwest China, were included in this study (refer to Fig. S4).

Our findings revealed that YEZV primarily infected *I. persulcatus* (31.4%) and *D. nuttalli* (10.5%) in northern China. Additionally, YEZV was detected in *R. microplus* (1.8%), a tick species with a global distribution (31). Conversely, JCTV exhibited high infection rates in *H. montgomeryi* (81.3%) and *H. longicornis* (11.1%), the latter having a widespread distribution in eastern Asia and recently reaching the USA (32). JCTV was also detected in *A. javanense* (7.7%) and *I. ovatus* (37.5%) from southern China. Notably, YEZV appeared to be predominantly distributed in Northern China, while JCTV showed a higher prevalence in southern China. Interestingly, both viruses were identified in Jiànchuān county in Yunnan province.

## DISCUSSION

In this investigation, we successfully isolated two new orthonairoviruses, namely, YEZV and JCTV, from ticks in China. Our findings trace YEZV infections back to 2012, with four tick-bite patients in northern China, with one patient displaying severe optic nerve disorder and CSF lymphocytic pleocytosis. The presence of YEZV RNA in this severe case underscores the diagnostic relevance of molecular testing. Additionally, we demonstrated that the vOTUs of both YEZV and JCTV exhibit DUB and deISGylase activity, although with a narrower spectrum than CCHFV during infection. These findings emphasize the need for continued surveillance and basic research to advance the diagnosis and treatment of nairoviral diseases.

Protein modification through ubiquitin and ubiquitin-like (Ubl) molecules plays a pivotal role in cell biology, particularly in anti-viral immune responses (11). ISG15, a Ubl molecule, is a key player in the host anti-viral response (33). DUBs including OTU have been uncovered as key regulators in crucial cellular processes and pathways such as nuclear factor-kappa B signaling (34). DUBs counteract the action of enzymes which catalyze ubiquitinylation, a process directed by a cascade comprising the activities of E1, E2, and E3 (35). The OTU domain is part of a larger superfamily of predicted cysteine proteases that possess a catalytic triad typically comprising cysteine, histidine, and aspartate residues (36). Otubain-1 and otubain-2 were the first two OTU proteins found to display *in vitro* DUB activity (37).

Some bacteria and numerous viruses have independently developed effector proteins to mimic the functions of host DUBs in order to increase infectivity, and pathogen-encoded DUBs may serve as drug targets for the treatment of infectious diseases (38). CCHFV encodes OTU domain-containing proteases regulating Ub- and ISG15-dependent innate immunity (12, 13, 16, 36). Our study and a previous one both indicated a high degree of vOTU diversity, with vOTU showing less than 25% amino acid identities within the family (39). The different vOTUs pose distinguishing structural features and distinct preferences for Ub and ISG15 (39, 40). The distinct vOTU from new orthonairoviruses such as Yánbiān orthonairoviral vOTU warrants further investigation.

The diversity of vOTU of orthonairoviruses suggests a potential relationship with viral host adaptation and pathogenesis. CCHFV, the most lethal to human, is endemic across large regions of sub-Saharan Africa, southeast Europe, and Asia. Up to now, although YEZV infections cause mild non-specific febrile illness to severe neurological symptoms based on this study and other reports (3, 8–10), no fatal case has been reported. While CCHFV’s vOTU exhibits broad deISGylase activity (16), our study indicates that YEZV and JCTV vOTUs demonstrate a more constrained deISGylase activity. These results prompt vOTUs to be considered potential virulence factors and explored for therapeutic purposes. In addition, unlike Ub, which is almost perfectly conserved among eukaryotes, ISG15 is highly divergent even among mammals (33). Deaton et al. illustrated that vOTUs from orthonairoviruses have clear preferences for ISG15s from certain species. It is suggested that there is some evolutionary pressure for tick-borne nairoviruses to possess vOTUs that are optimized to ISG15 of susceptible vertebrate species (41). The vOTU of JCTV also had deISGylating activity to human ISG15, and this virus was circulating in southern China, indicating more active surveillance should be carried out.

It is always interesting to find that the emergence of some new viruses can be traced back much earlier. For example, SFTSV, another pathogenic tick-borne virus, was first discovered in Henan Province (42), China, in 2009 and has been traced back to Jiangsu Province, China in 1996 (43). YEZV, identified as a novel orthonairovirus, was first reported in patients experiencing acute febrile illness after tick bites in Hokkaido, Japan, in 2019 and 2020 (3). Our retrospective survey identified four additional cases, indicating YEZV’s presence in the region since at least 2012. Clinical presentations of YEZV infections appear non-specific, primarily manifesting as febrile illness. Antibodies to YEZV were found in Hokkaido shika deer and raccoons, suggesting potential wildlife reservoirs (3). Our study expands the understanding of YEZV’s vector distribution, with predominant infection in *I. persulcatus*, *D. nuttalli*, and *R. microplus* in the field, warranting future investigations into the virus’s life cycle and competent vectors.

In conclusion, our work unveils substantial genomic diversity among vOTUs from different orthonairoviruses, suggesting the potential pathogenicity of numerous orthonairoviruses in humans, posing a significant public health risk. A comprehensive understanding of orthonairoviruses is crucial for physicians to recognize emerging nairoviral diseases and for researchers to translate insights from vOTU domains into therapeutic development. Ongoing surveillance efforts and continuous research will be instrumental in addressing the challenges posed by these emerging viruses.

## Data Availability

The sequences of L, M, and S segments of Jiànchuān tick virus and YEZV have been deposited in the GenBank with the accession numbers ON872594, PP708717, PP708718, and PP708714-716.

## SUPPLEMENTAL MATERIAL

Supplemental material jvi.01727-24-s0001.docx Figures S1 to S4 and Tables S1 and S2.

ASM does not own the copyrights to Supplemental Material that may be linked to, or accessed through, an article. The authors have granted ASM a non-exclusive, world-wide license to publish the Supplemental Material files. Please contact the corresponding author directly for reuse.
