# Supplementary material for: Characteristics of viral ovarian tumor domain protease from two emerging orthonairoviruses and identification of Yezo virus human infections in northeastern China as early as 2012
Source: J Virol. 2024 Dec 31;99(2):e01727-24. doi: 10.1128/jvi.01727-24 (PMC11852922; doi:10.1128/jvi.01727-24)
Supplement: Supplemental material — Figures S1 to S4 and Tables S1 and S2. [file jvi.01727-24-s0001.docx]

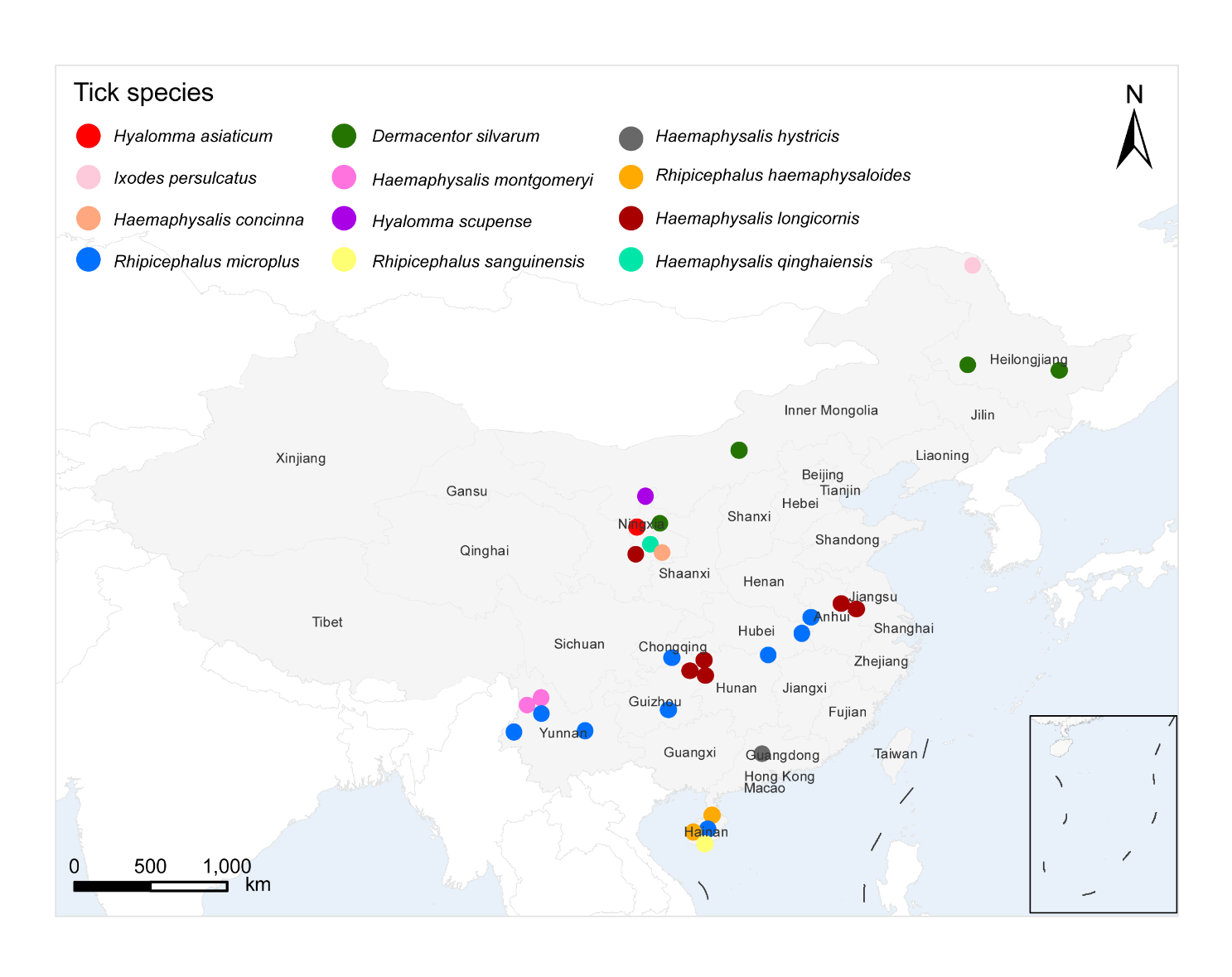


**Supplemental Figure 1. Map of tick sample sites where samples were used to isolate orthonairoviruses.**

400 archived tick samples from 19 sites across 10 provinces were detected for orthonairovirus isolation. The points of different colors represent different tick species.

**
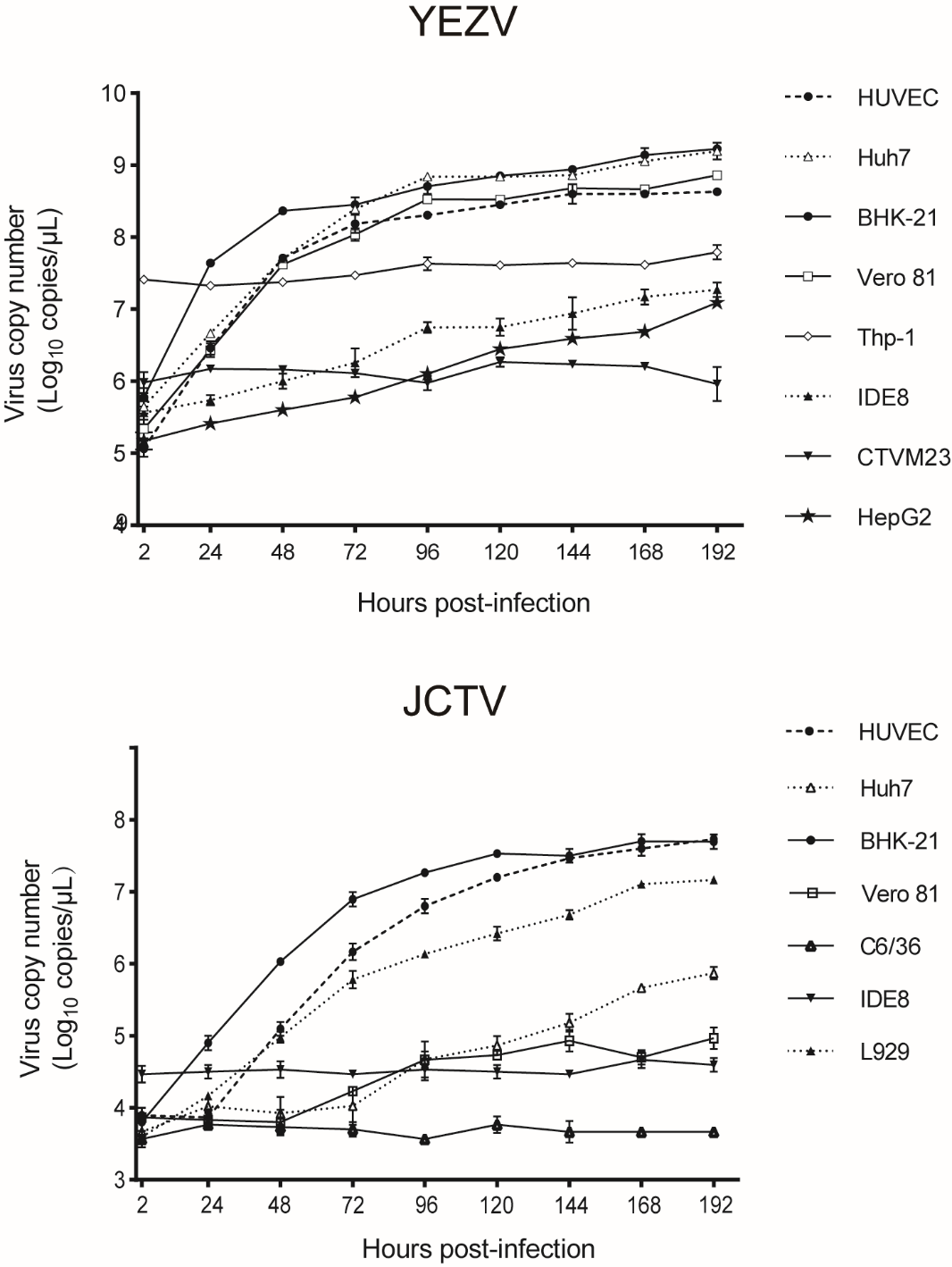
**

**Supplemental Figure 2. Growth curves of YEZV and JCTV in different cells over 192h.**

The cultures of Vero81, BHK21, Huh7, HUVEC, C6/36, IDE8, CTVM23, HepG2, L929, and Thp-1 were infected with YEZV or JCTV over 192h. Error bars represent the standard deviation of the mean.


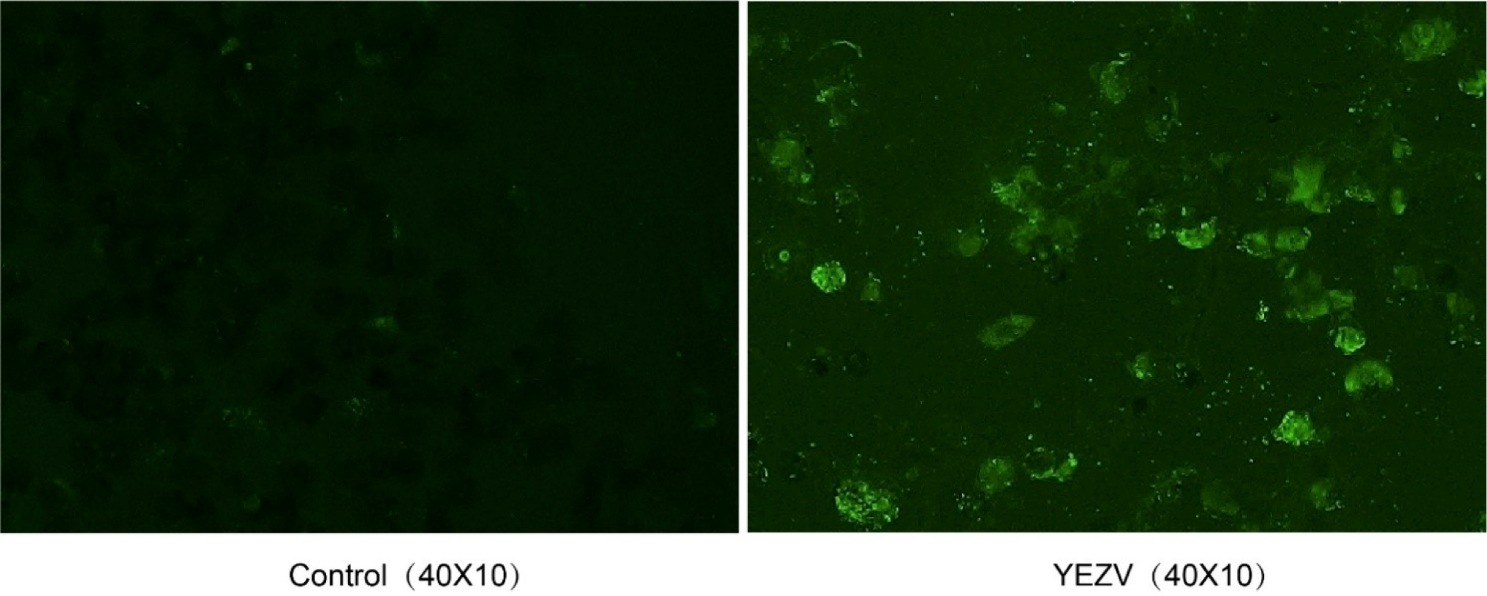


**Supplemental Figure 3.** **Indirect** **Immunofluorescence Assay of YEZV in serum of tick bite patients.**

Serum samples of patients were detected seroconversion of IgG antibodies against YEZV by IFA assays.

**
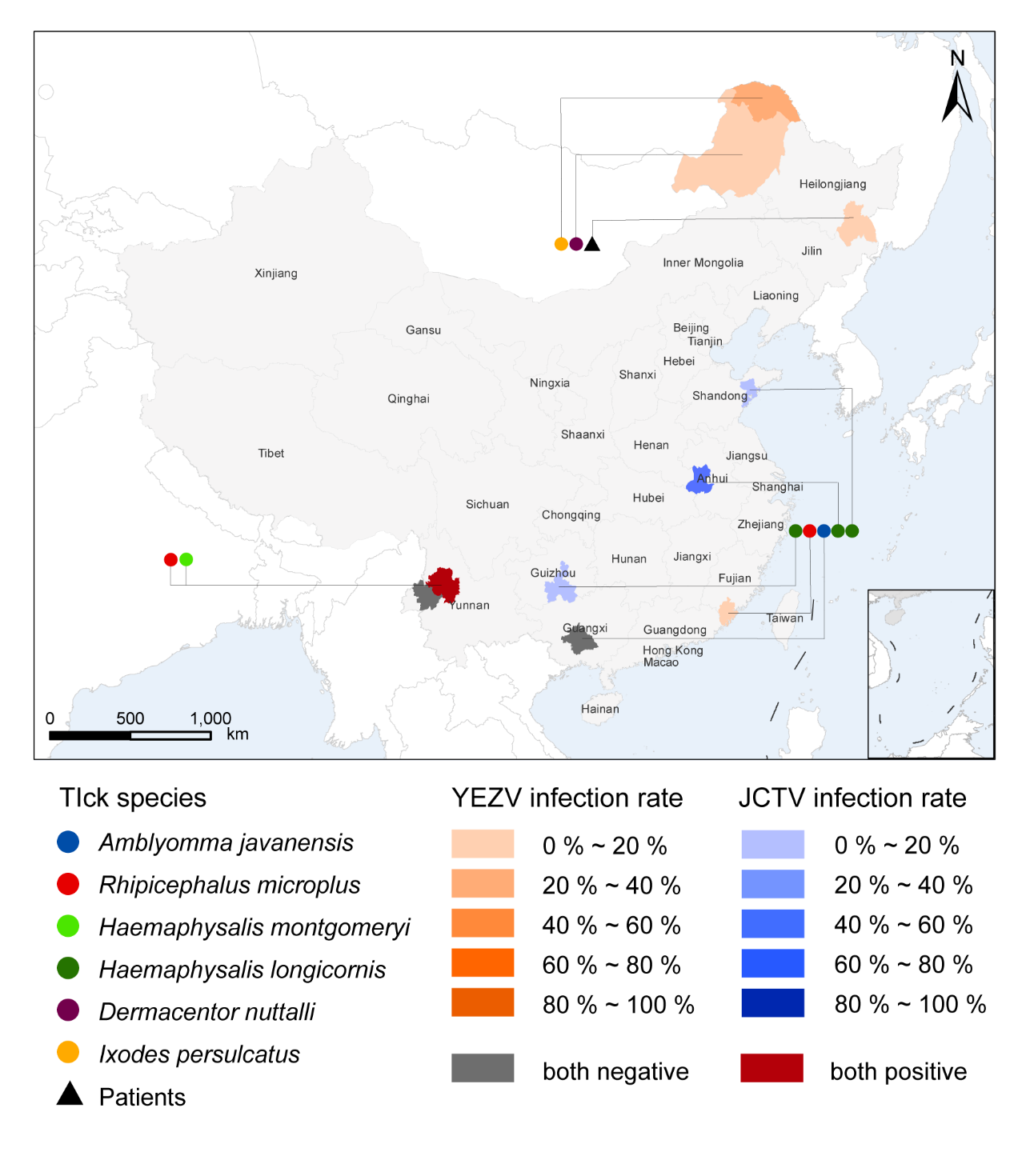
Supplemental Figure 4.** **Maps of tick samples for JCTV and YEZV detection.**

Additional 868 adult ticks sourced from 9 sites across 8 provinces were individually tested using qRT-PCR with specific primers for YEZV and JCTV. Tick species and patients are also shown on the map, denoted by different shapes and colors. Infection rates of these sites are indicated by different colors.

| **Supplemental Table 1. Primers of 6 known pathogenic orthonairoviruses and 3 new orthonairoviruses** | | | |
| --- | --- | --- | --- |
| **Virus** | **Gene length (bp)** | **Primer** | **Sequence** |
| Yezo virus | 220 | YEZO-F | ATTTAGGAGTTTAGCGATGC |
|  |  | YEZO-R | CCTGGCTGGTTTCACATC |
| Nairobi sheep disease virus | 483 | NSDV-F | TTACTTGGTCCGTCACCC |
|  |  | NSDV-R | ATTGCCATTCCAGTTTCC |
| Sōnglǐng virus | 497 | SGLV-F | AACCAAAGTTAGTGACCACCCT |
|  |  | SGLV-R | CTGCCTGCTGCTCATCCT |
| Tǎchéng tick virus 1 | 244 | TCTV1-F | GGCAGAAGAAGTCAATAAG |
|  |  | TCTV1-R | CAGGGTTTCAGGAATGTA |
| Tamdy virus | 446 | TMDY-F | GCTGTCCCTGGTCAATAC |
|  |  | TMDY-R | AAGCCTTTCACCTTCTGT |
| Běijí nairovirus | 238 | BJNV-F | AGGACCCATCTGAACTGA |
|  |  | BJNV-R | GTGGATATTGCCCTAACA |
| South Bay virus | 277 | SBV-F | GCACCACTGCCATTCTAC |
|  |  | SBV-R | GTCCCATGCCTTTCATTT |
| Bole tick virus 4 | 254 | BLTV4-F | ACGTCACCGGCAGAAAGA |
|  |  | BLTV4-R | TGTTGTTGAAGAATGGCACAT |
| Jiànchuān tick virus | 93 | JCTV-F | AGTGGAGACAAACATGGAAGAA |
|  |  | JCTV-R | TCAGAGTTCCTGACACGAGTA |
|  |  | JCTV-P | TGCACAGTCTTCAGGAAGGTGCTC |

| **Supplemental Table 2. M segment-specific probe sets of JCTV and YEZV** | | | |
| --- | --- | --- | --- |
| **Primer** | **Probe Sequence** | **Primer** | **Probe Sequence** |
| YEZO_M_1 | ccctacactgcatgattaac | JCTV_M_1 | catctcctaacatgcatgtt |
| YEZO_M_2 | cacactgatgccaataacca | JCTV_M_2 | agggagaccatgcacagaaa |
| YEZO_M_3 | gtcttctcaggtaatgtctt | JCTV_M_3 | agctaattcaccagaatcct |
| YEZO_M_4 | cggtggaagtcttcatagtt | JCTV_M_4 | gaggcgaggttacaaaaggc |
| YEZO_M_5 | ttggaaaggtttccggagta | JCTV_M_5 | catcttcatacttatcggct |
| YEZO_M_6 | cagagttgtagtaagctgct | JCTV_M_6 | tttttggaggaattggcagc |
| YEZO_M_7 | ttacagcctcttgcatcaaa | JCTV_M_7 | agagcaccgaagaactttgc |
| YEZO_M_8 | gtcaatagatacatgctgcc | JCTV_M_8 | aaattctgtgcacggctcaa |
| YEZO_M_9 | attggatacacctttccatt | JCTV_M_9 | ttctatgctgctgatgatgt |
| YEZO_M_10 | agagattgcacagttctctc | JCTV_M_10 | tgatgcacctacaacctatg |
| YEZO_M_11 | gtttttttgtttgtctcacc | JCTV_M_11 | catcaggatgacacatccag |
| YEZO_M_12 | gtagtttcctggaaaacgct | JCTV_M_12 | catgtgaaaccagaagggct |
| YEZO_M_13 | aatgatgactctggggcatg | JCTV_M_13 | tgtcaactacttcttccaca |
| YEZO_M_14 | agttatgcatcacagtagct | JCTV_M_14 | caacgtccagtctctatttg |
| YEZO_M_15 | tagacttcctttaggagcag | JCTV_M_15 | gtaagaatggcagacggcag |
| YEZO_M_16 | ccgtcaggacatgttacaag | JCTV_M_16 | tccaaagcacgaccattttg |
| YEZO_M_17 | attggtgtgcagtactatgt | JCTV_M_17 | tcatcaggggtaactctaca |
| YEZO_M_18 | agattgcaggcttacctaag | JCTV_M_18 | ttccaagtgggaacttgact |
| YEZO_M_19 | aatgcaatcctaccaatgct | JCTV_M_19 | ctgggagtgaagacactctt |
| YEZO_M_20 | tgcttgtccattctgaagat | JCTV_M_20 | cagatttctgtactccactg |
| YEZO_M_21 | cagtaaggacaggatccaga | JCTV_M_21 | caaggaaatcaggcgtgtct |
| YEZO_M_22 | ccttcaacttggaaaggctg | JCTV_M_22 | gggaaatcagctctcctaag |
| YEZO_M_23 | actactacccactgaattct | JCTV_M_23 | agtgatgacacttgaggctg |
| YEZO_M_24 | actggctggcaaacttcaag | JCTV_M_24 | tttccaagtgcaaagcttct |
| YEZO_M_25 | cgattcttgaattgggcagg | JCTV_M_25 | gatggctgtaaggaagcttc |
| YEZO_M_26 | gaccttttgactatgtggtg | JCTV_M_26 | acctttggactgaaggacac |
| YEZO_M_27 | agctacgggcagagaaagta | JCTV_M_27 | tgtctctgatactgctgtac |
| YEZO_M_28 | caatgtctagatctctgggg | JCTV_M_28 | caagaagccatgaccatcag |
| YEZO_M_29 | agggtctagtcttagtatgg | JCTV_M_29 | aataccactggcaaaggcat |
| YEZO_M_30 | agatgtttctgctctcagag | JCTV_M_30 | caggatggatgacactaggg |
| YEZO_M_31 | aagttcccaactttctgttg | JCTV_M_31 | acatggagactcttcacact |
| YEZO_M_32 | caaccacacttttcaggaca | JCTV_M_32 | tatggtacctttttggctat |
| YEZO_M_33 | tgagagctgcatgtatgact | JCTV_M_33 | ggactgcaagggtgaatgta |
| YEZO_M_34 | aagggtttgtcaatgtctgc | JCTV_M_34 | cttatagcaggtttggggaa |
| YEZO_M_35 | ccatttagtcaagatccagt | JCTV_M_35 | aaagtgcaatgtcctgcatc |
| YEZO_M_36 | gacgactgccatgttcaaac | JCTV_M_36 | acgcatgataaaccagttcc |
| YEZO_M_37 | cctcctagagatatagttgt | JCTV_M_37 | actctccatcaatccaagag |
| YEZO_M_38 | atctttatgatttcctgggg | JCTV_M_38 | gaagggaagcccaaagaagc |
| YEZO_M_39 | atcattccaattggcttcac | JCTV_M_39 | acagtcaatagagccaggat |
| YEZO_M_40 | gtattgaagcacagttcctt | JCTV_M_40 | ttgctgatttgaccgaagga |
| YEZO_M_41 | ttccagaggttttcaaacgc | JCTV_M_41 | tccctaggtggaacaatttg |
| YEZO_M_42 | gttggtgatccatctctaat | JCTV_M_42 | taaaaaacgtgggtgccggt |
| YEZO_M_43 | agcactgtctttgactctag | JCTV_M_43 | gaattcatcgcaggtgatcg |
| YEZO_M_44 | tagagtggtttctgaggcaa | JCTV_M_44 | aggaaggcaaggtgctgaat |
| YEZO_M_45 | aacttgatgcatcctctgac | JCTV_M_45 | ctctttcatgtccattatct |
| YEZO_M_46 | tctggaggctgaagcttaac | JCTV_M_46 | aggctggaatgaagcagttc |
| YEZO_M_47 | aggcaagacaagaacccttt | JCTV_M_47 | atggggaacatgacgaacct |
| YEZO_M_48 | gcttaaaaggttgggtccaa | JCTV_M_48 | aggagcctgtatatactcat |
